# Modulating the PI3K Signalling Pathway in Activated PI3K Delta Syndrome: a Clinical Perspective

**DOI:** 10.1007/s10875-023-01626-0

**Published:** 2023-12-27

**Authors:** Lucinda J. Berglund

**Affiliations:** 1 Faculty of Medicine, University of Sydney, Sydney, NSW Australia; 2 Department of Immunopathology, Westmead Hospital, NSW Health Pathology, Westmead, Sydney, NSW Australia

**Keywords:** Activated PI3K delta syndrome, APDS, leniolisib, sirolimus

## Abstract

Activated phosphoinositide-3-kinase (PI3K) δ syndrome (APDS) is an inborn error of immunity characterised by immune dysregulation. Since the discovery of genetic mutations resulting in PI3Kδ overactivation, treatment of APDS patients has begun to focus on modulation of the PI3K pathway in addition to supportive therapies. The mTOR inhibitor sirolimus has been used effectively for some clinical manifestations of this condition, however the arrival of specific PI3Kδ inhibitor leniolisib has shown promising early results and may provide a more targeted approach. This review summarizes key aspects of PI3K pathway biology and discusses potential options for nuanced modulation of the PI3K pathway in APDS from a clinical perspective, highlighting differences from PI3K inhibition in haematological malignancies.

## Introduction

Activated phosphoinositide-3-kinase (PI3K) δ syndrome (APDS) is an inborn error of immunity characterised by dysregulation of the immune system. Clinical manifestations vary from lymphoproliferation, enteropathy and cytopenias, to recurrent infections, often from a young age, and an increased risk of lymphoma. A recent European study described respiratory infections as the most common clinical manifestation of APDS affecting 92% of individuals, with bronchiectasis in 50%, benign lymphoproliferation in 86%, enteropathy in 35%, cytopenias in 19% and other autoimmune conditions occurring less frequently [1]. APDS is biochemically characterised by overactivation of the PI3K-Akt-mechanistic target of rapamycin (mTOR) pathway. In APDS1, activating mutations in the *PIK3CD* gene encoding the p110δ catalytic subunit of PI3Kδ lead to intrinsically increased PI3Kδ activity by disrupting inhibitory contacts between p110δ and the p85α regulatory subunit [2, 3]. In APDS2, heterozygous variants in *PIK3R1* encoding p85α reduce inhibition of PI3Kδ, again resulting in overactivation of the PI3K-Akt-mTOR pathway [4, 5]. A third condition, APDS-Like (APDS-L), arises from heterozygous loss of function mutations in ubiquitously expressed phosphatase and tensin homolog (*PTEN*), leading to the PTEN hamartoma tumour syndrome [6]. This condition has a wide range of clinical manifestations, with immunodeficiency and features of immunodysregulation reported in some patients [7]. PTEN converts phosphatidylinositol-3,4,5-trisphosphate (PIP_3_) back to phosphatidylinositol-4,5-bisphosphate (PIP_2_), terminating the signal triggered by PI3K activation. Hence loss of PTEN function can also result in PI3K pathway overactivation [7]. This mini-review discusses key clinical manifestations of APDS resulting from dysregulated PI3K pathway hyperactivity and focuses on the specific clinical management options for patients with APDS1 and APDS2 by considering the effects of modulating the PI3K-Akt-mTOR pathway.

The PI3K pathway is essential across species for a range of cellular functions. In immune cells, this pathway regulates cell growth, differentiation, activation and survival [8]. Class IA PI3Ks are essential for signalling downstream of the B and T cell receptors, Toll-like receptors, cytokine receptors, co-receptors and adhesion molecules in B and T lymphocytes and myeloid cells [8]. p110δ is the main catalytic isoform in leucocytes [9]. PI3K is activated by phosphorylation of canonical YxxM motifs in the cytoplasmic domain of transmembrane receptors and adaptor proteins, such as CD19, CD28, ICOS and B cell adaptor protein (BCAP), which are required for effective signalling downstream of the B and T cell receptors [10].

**Fig. 1:**
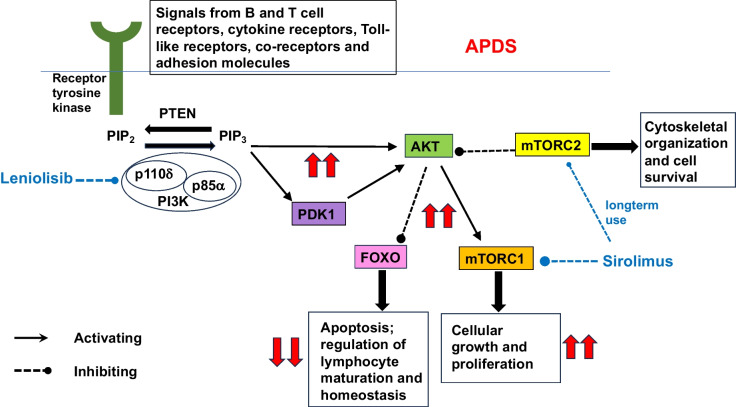
Key components of the PI3K-Akt-mTOR signalling pathway. The effects of increased signalling through this pathway in APDS are shown in red. The sites action of leniolisib and sirolimus are shown in blue

The immune dysregulation in APDS is characterised by an excess of immature B cells and senescent T cells and a deficiency of functional immune cells [3, 5, 11]. p110δ is required to variable degrees at different stages of lymphocyte development [12]. Physiologically, the PI3K pathway is up- or down-regulated at developmental points to allow Forkhead Box O (FOXO)-dependent signalling, which is essential for the regulation of lymphocyte maturation and homeostasis [12]. Constitutive activation of PI3K signalling in APDS prevents this nuanced response (Fig. 1). The clinical manifestations of APDS, however, are heterogenous, with some patients demonstrating a more severe phenotype, and others being more mildly affected, even within the same family. This suggests that the PI3K pathway function is variably altered despite the same genetic variant [13, 14].

In APDS1, PI3Kδ hyperactivation results in impaired B cell maturation with accumulation of pre-BII cells in bone marrow, increased transitional B cells in peripheral blood and reduced memory B cells [13, 15]. Immunoglobulin (Ig) class-switching is also impaired, characterised by elevated levels of serum IgM, but reduced IgG and IgA, and reduced IgG^+^ and IgA^+^ memory B cells [13, 15]. Antibody responses to protein and polysaccharide vaccinations are also reduced [2, 16]. Similar B cell developmental impairment has been described in APDS2, with increased transitional and reduced mature B cell subsets and reduced class-switching to IgG, however pro-B cells rather than pre-BII cells accumulated in the bone marrow of two APDS2 patients [14, 17]. Clinically, these humoral impairments manifest as recurrent bacterial sinopulmonary infections from an early age, with *Haemophilus influenzae* and *Streptococcus pneumoniae* often implicated, sometimes culminating in bronchiectasis [13, 14].

T cells are also affected in APDS1, with reduced numbers and proportions of circulating CD4^+^ T cells, particularly naïve CD4^+^ T cells, whereas central and effector memory CD4^+^ T cells are increased [18]. Circulating T follicular helper (Tfh) cells are also increased, however cytokine secretion by Tfh and memory CD4 + T cells is aberrant [18]. This CD4^+^ T cell dysregulation leads to impaired provision of help to B cells, adding to the clinical features of humoral immunodeficiency. Naive CD8^+^ T cells are also reduced, whereas effector memory CD8^+^ and terminally differentiated CD8^+^ T cells expressing CD45RA are increased [19, 20]. The CD8^+^ T cell compartment appears exhausted and senescent [3, 20]. NK cell function is also impaired in APDS1 [21]. Similar CD4^+^ and CD8^+^ T cell abnormalities have been described in APDS2 [14, 17]. Clinically, these CD8^+^ T cell and NK cell abnormalities predispose to persistent, severe or recurrent herpesvirus infections, such as Epstein-Barr virus (EBV) and cytomegalovirus [13]. Lymphadenopathy may result from an accumulation of proliferating B cells and Tfh cells [13, 14], and an overabundance of effector T cells with an exaggerated proliferative response to antigen [3]. EBV-driven lymphoproliferation similar to post-transplant lymphoproliferative disease can occur [13]. EBV and chronic antigenic stimulation may also contribute to an increased malignant transformation of B cells [22], leading to an increased risk of lymphoma, which has been reported in 13% of APDS1 and 28% of APDS2 [13, 14, 23].

The delicate balance of PI3Kδ activity under physiological conditions is further highlighted by findings from preclinical and clinical studies demonstrating equally profound immune dysregulation resulting from PI3Kδ pathway loss of function [24]. Genetic mouse models with impaired PI3Kδ function, such as *Pik3cd*^*−*^*/*^*−*^ mice, develop immune dysregulation, poor vaccine responses and enterocolitis, most likely due to reduced regulatory T cell function [25–27]. These findings are confirmed in patients with homozygous biallelic deletion or loss-of-function mutations in *PIK3CD,* which demonstrate various forms of immunodeficiency, characterized by a profound block in B cell development and a range of immune dysregulatory manifestations including sinopulmonary infections, autoimmune enterocolitis, autoimmune hepatitis and arthritis [28–32]. Biallelic loss of *PIK3R1* (p85α) has also been reported and leads to a block in B cell development [33].

These findings have important implications for any pharmacological intervention in the PI3Kδ pathway.

## Therapeutic PI3K Pathway Modulation

Identification of the pathogenic variants in APDS has guided the therapeutic use of medications which alter PI3K pathway function in this condition. Haematopoietic stem cell transplantation (HSCT) remains the only curative treatment, but carries a significant risk of mortality, evidenced by 5 deaths reported amongst 29 APDS patients receiving HSCT in a recent study [1]. HSCT in APDS is also frequently complicated by graft failure, graft instability and poor graft function [34]. Other supportive therapies, such as gammaglobulin replacement and prophylactic antibiotics, reduce bacterial infections but have less effect on herpesvirus infections, lymphoproliferation or lymphoma, and autoimmune manifestations [35]. Corticosteroids and rituximab can help control cytopenias, autoimmunity and lymphoproliferation [35], but also cause adverse effects, including an increased risk of infection and hypogammaglobulinemia. Use of rituximab in chronic EBV infection in APDS has also been described [36]. The hope of novel therapies is that they will provide a much more precisely targeted intervention in APDS, treating the whole spectrum of clinical manifestations with limited and acceptable toxicity.

## mTOR Inhibition

### Mechanism of Action and Efficacy in APDS

Sirolimus, also known as rapamycin, is an mTOR inhibitor acting downstream of PI3K, including PI3Kδ but also other isoforms. The effects of sirolimus on mTOR signalling are complex. Sirolimus forms a gain-of-function complex with the FK506-binding protein (FKBP12), which binds and inhibits the mTOR complex 1 (mTORC1) [37]. The mTORC1 pathway integrates signals from a wide range of intracellular and extracellular factors beyond PI3K input, including growth factors, cellular stress, DNA damage, energy status, oxygen and amino acids, and downstream controls protein and lipid synthesis and autophagy [37]. Conversely, mTORC2 assembly and signalling is reduced only in some cell types after long-term sirolimus treatment [37]. mTORC2 activates Akt and serum/glucocorticoid-regulated kinase 1 (SGK1) and plays a role in actin cytoskeletal organisation and cell survival [38].

Sirolimus has demonstrated utility in APDS in retrospective registry and case series analysis, particularly in reducing lymphadenopathy and splenomegaly [1, 13, 14, 23, 39]. Decreased requirement for corticosteroids and improvement in lymphocyte subsets have also been observed, even at low trough drug levels [39]. Enteropathy and cytopenias may respond less well to sirolimus, although the mechanism underlying these clinical improvements has not been defined [23]. The pathogenesis of cytopenias and enteropathy in APDS has been not yet been determined, but may depend less on overactive signalling via mTORC1 than other downstream pathways, including FOXO. Pharmacokinetic issues may also play a role in the efficacy of sirolimus in treating these manifestations, as outlined below. At a cellular level, sirolimus can partially restore NK cell function in APDS [21]. Improved T cell senescence pattern with increased naïve T cell percentages [3, 39], and normalisation of CD8^+^ T cell counts [3] have also been observed. There are no published data suggesting either a positive or negative effect of sirolimus on the frequency or severity of infections. Sirolimus has also been shown to improve immunological parameters and gastrointestinal symptoms in a patient with APDS-L [40].

### Pharmacokinetics

Treatment with sirolimus is not always straightforward. A wide variety of factors influence the pharmacodynamic and pharmacokinetic response to sirolimus, including age, weight, hepatic function, and genetic polymorphisms influencing expression of cytochrome P450 3A4 (CYP3A4), cytochrome P450 3A5 (CYP3A5) and p-glycoprotein (PgP) [41, 42]. Accordingly, therapeutic drug monitoring using trough sirolimus concentrations is considered standard of care [43], given the narrow therapeutic window and risks of toxicity. Oral bioavailability is poor, with extensive first-pass metabolism, a large volume of distribution and considerable distribution amongst red blood cells (94%), lymphocytes (1%) and granulocytes (1%) [43]. Although only 3.1% is distributed within whole blood, sirolimus is 40% bound to lipoproteins in a concentration-dependent manner, hence therapeutic drug monitoring of whole blood is preferred [43]. Sirolimus is metabolised in the liver and small intestinal microsomes by CYP3A4 and CYP3A5 [44], leading to potential drug interactions with CYP3A4 inducers and inhibitors, including antibiotics, azole antifungals, calcium channel blockers, grapefruit juice and St John’s wort. Circadian and seasonal variations in CYP3A4 activity have also been reported [45, 46]. Sirolimus is transported by the multidrug resistance gene product pump PgP, expressed widely in enterocytes [41, 44]. It is possible that the pharmacokinetics of sirolimus, including poor oral bioavailability, dependence on PgP expression in enterocytes for transport, and metabolism via cytochrome activity in enterocytes as well as the liver, might reduce the absorption and activity of sirolimus in the context of enteropathy. Likewise, the extensive sequestration of sirolimus in red blood cells might mean that the pharmacokinetics of sirolimus are altered in the patients with significant anaemia or other cytopenias. Further study of the pharmacokinetics of sirolimus in patients with these clinical manifestations might answer these questions.

Reliance on therapeutic drug monitoring may not be adequate, however, as the correlation between the area under the curve (AUC) and sirolimus trough concentration is inconsistent, particularly in paediatric patients [47, 48]. If such variability can be demonstrated in children after renal and stem cell transplantation, it is likely that such variability also applies in APDS, given the widely differing clinical phenotypes and degree of PI3K pathway hyperactivation. Model-informed precision dosing of sirolimus in children has been suggested [41], and would likely also be of benefit in APDS. Furthermore, the target trough concentration range of 10–15 ng/ml recommended for immunosuppression in solid organ transplantation may be higher than required in APDS [39], where a more subtle modulation of the PI3K pathway is the aim. Optimal sirolimus trough concentrations in APDS have not yet been determined.

### Adverse Effects

Adverse effects of sirolimus in general include hyperglycaemia, dyslipidaemia, cytopenias, nephrotoxicity, stomatitis and skin eruptions, poor wound healing and pneumonitis [38]. The increased risk of new onset diabetes mellitus is likely due to altered insulin signaling via mTOR inhibition, insulin resistance and dysfunctional insulin secretion [38]. New onset diabetes has not yet been recorded in APDS patients treated with sirolimus, although some of the risk of hyperglycaemia is mediated via mTORC2 after long-term sirolimus therapy. Thus, monitoring APDS patients on sirolimus for longer periods will be required to assess the long-term risk of hyperglycaemia in this population. Likewise, dyslipidaemia occurs in 40–75% of solid organ transplant recipients receiving sirolimus [49], affecting both triglycerides and cholesterol, and although this complication has not been recorded in APDS, long-term data are not yet available in this population.

Sirolimus seems to be tolerated more easily in APDS, compared to other indications such as solid organ transplantation, where discontinuation rates due to adverse effects in clinical trials range from 20–40% [38, 50]. Perhaps improved tolerance arises because the PI3Kδ-Akt-mTOR pathway is already overactive in APDS, hence sirolimus may not reduce mTORC1 signalling below physiological levels, in leucocytes at least. Sirolimus, however, also targets mTORC1 downstream of other PI3K isoforms. In an analysis of 26 patients receiving sirolimus for APDS, 2 patients required complete cessation of sirolimus due to severe headaches, anorexia and/or renal toxicity, and 3 patients paused sirolimus due to aphthous ulcers, liver toxicity and/or renal toxicity, but were able to restart [23]. Recurrent and severe aphthous stomatitis has also been reported after sirolimus in a single patient with APDS2 [51]. Further data will be required to determine the long-term safety of sirolimus in APDS, particularly pertaining to metabolic complications which may become more relevant with increasing longevity.

In summary, sirolimus is a useful treatment in some patients with APDS, but is limited by complex pharmacokinetics, the need for ongoing trough level monitoring, and limited capacity to improve cytopenias, enteropathy and infections.

## Newer mTOR Inhibitors

Other rapalogs, such as everolimus and temsirolimus, have not been studied in APDS. Everolimus has shown efficacy in other inborn errors of immunity causing immunodysregulation, such as CTLA4 haploinsufficiency [52]. Everolimus, a 40-O-(2-hydroxyethyl) derivative of sirolimus, also inhibits mTOR signalling, but more potently inhibits mTORC2, and its pharmacokinetics differ from sirolimus [53]. Thus, the efficacy and/or side effect profile may vary in APDS, warranting further study. Second generation mTOR inhibitors have been developed for oncology trials and include sapanisertib, an adenosine-triphosphate-competitive mTOR kinase inhibitors which suppresses mTORC1 and mTORC2 [54, 55]. Third generation mTOR inhibitors, such as RapaLink-1, combine sirolimus with a second generation mTOR kinase inhibitor [55]. Neither second nor third generation mTOR inhibitors have been described for use in APDS, and although greater efficacy in killing cancer cells may be preferable in oncology trials, APDS is likely to require more subtle degrees of regulation of the mTOR pathway, specifically downstream of PI3Kδ, with over inhibition of the PI3K-Akt-mTOR pathway in general potentially increasing toxicity, particularly in long-term use.

## PI3K Inhibition

The PI3K pathway is dysregulated in a wide range of human cancers, including breast cancer, colorectal cancer and haematological malignancies [56]. Genetic mutations can affect many signalling molecules in this pathway, the most common being *PIK3CA*, which encodes PI3Kα, and *PTEN*.[57] The p110δ subunit of PI3Kδ, encoded by *PIK3CD* and affected in APDS1, is primarily expressed by haematopoietic cells, and is therefore most relevant as a potential target in haematological malignancies. An extensive number of PI3K inhibitors have been evaluated for use in oncology, including isoform-specific inhibitors, pan-PI3K inhibitors, and dual PI3K-mTOR inhibitors. Due to the ubiquitous and vital expression of the PI3K pathway in a range of cells, the use of many pan-PI3K inhibitors has been limited by toxicity, particularly gastrointestinal, hepatic and cutaneous, and hyperglycaemia, and many have also shown limited anticancer efficacy [56]. The adverse effects of iatrogenic overinhibition of the PI3K pathway not unexpectedly resemble the clinical manifestations of deletion or loss-of-function mutations in *PIK3CD* described above, specifically enterocolitis and hepatitis [28–32].

These results suggest that pan-PI3K inhibition could be expected to be excessively toxic for the long-term use required in APDS. Historically, use of PI3K pathway inhibitors in cancer therapy has applied mainly to patients with advanced cancers and limited life expectancy, hence strategies to reduce side effects focused more on short-term rather than long-term complications, with an acceptance of some degree of toxicity in the short term in the interest of extending life [58]. As increasing numbers and types of PI3K pathway inhibitors are approved for cancer therapy, and as patients survive longer, metabolic complications such as hyperglycaemia might become more relevant. In contrast, patients with APDS are likely to require more subtle PI3K pathway suppression for a prolonged period, and thus both short- and long-term toxicity are highly relevant.

## PI3Kδ-Specific Inhibition

Leniolisib, an oral small molecule inhibitor of p110δ, has been shown to cause dose-dependent suppression of PI3K pathway hyperactivation, measured as phosphorylation of Akt/S6 in cell lines, and improved immune dysregulation in six patients with APDS1 [59]. These results were confirmed in a phase 3 randomised, blinded, placebo-controlled study of 31 patients with APDS 1 and 2, with significant reduction in lymphadenopathy, spleen size and serum IgM, improved cytopenias and increased circulating naïve B cells and other lymphocyte subsets in patients receiving leniolisib 70 mg twice daily for 12 weeks compared to placebo [60]. Practically, immunoglobulin replacement was reduced and even ceased in some patients receiving leniolisib, and subjective improvements in patient wellbeing, activity and energy levels have also been reported [59, 60]. Leniolisib appeared to be well tolerated so far, with mild adverse effects including non-severe, transient neutropenia in the absence of infection, and transient alopecia in two patients [60]. Late-onset immune-related adverse effects have not yet been reported, albeit a relatively new medication. The lack of severe adverse effects may reflect the fact that the recommended dose of 70 mg twice daily reduced PI3K/Akt pathway activity to within the normal range of unstimulated phosphorylated Akt levels in healthy controls. Although there are no trials directly comparing leniolisib to sirolimus, leniolisib seems promising in APDS, providing more targeted PI3Kδ regulation with limited toxicity [61]. Conceptually, directly targeting PI3Kδ in a condition arising from dysregulation of the pathway at that point seems likely to be a cleaner therapeutic option than inhibition of a downstream target, such as mTORC1.

Other PI3Kδ inhibitors have proven less successful in the treatment of APDS. In an open-label trial of five patients over 12 weeks, nemiralisib, an inhaled PI3Kδ inhibitor, did not reduce PIP_3_ in sputum, nor result in any significant changes in lymphocyte subsets [62]. Seletalisib, an oral PI3Kδ inhibitor, appeared to improve immune dysregulation but caused serious adverse effects, particularly colitis and drug-induced liver injury [63]. The PI3Kδ-specific inhibitor idelalisib has shown efficacy in B-cell lymphoproliferative malignancies including chronic lymphocytic leukaemia and follicular lymphoma [64–66]. Idelalisib has been associated with colitis, hepatitis and pneumonitis, which can be severe [67], requiring dose interruption, dose reduction, corticosteroids or drug discontinuation [67].

**Fig. 2:**
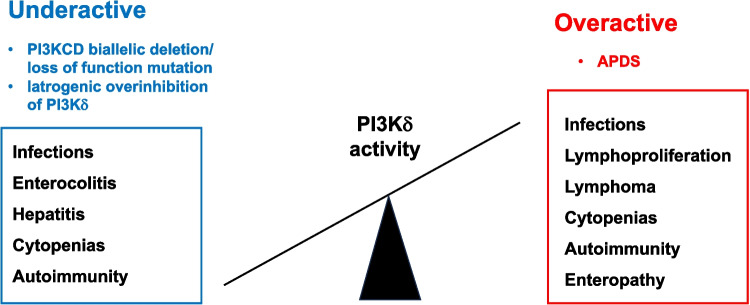
Causes and consequences of over- and under-activation of PI3Kδ

Excessive PI3Kδ inhibition may not be without risk (Fig. 2). PI3Kδ-specific inhibitors, including idelalisib, have been shown to increase genomic instability in normal and neoplastic B cells, through enhanced expression of activation-induced cytidine deaminase (AID) and subsequent increased somatic hypermutation and chromosomal translocation frequency [68]. As some of these effects are off-target (affecting genes other than immunoglobulin genes), this may increase the risk of oncogenic mutations or translocations, even in the absence of reduced B-cell proliferation [68]. However, reduced AID expression has been observed in APDS and contributes to defective class-switch recombination [15]. Similar findings have been shown in murine *Pik3cd*^*GOF*^ B cells, with normalisation of *Aicda* expression with leniolisib in vitro.[15] These results suggest that leniolisib may normalise, rather than exaggerate, AID expression in APDS, thus genomic instability may be less of a risk. Nevertheless, as APDS patients may require decades of treatment, avoiding iatrogenic over suppression of PI3Kδ may be essential to avoid the potential for haematological malignancy. Preliminary studies of leniolisib regarding longer term safety appear promising, with no new features of lymphoproliferation in 37 APDS patients studied for up to 5 years, median 2 years [69]. Furthermore, individuals with APDS already have an increased risk of lymphoma [13, 14], so the effects of PI3Kδ inhibition on lymphoma risk long term will need to be carefully assessed.

## Akt and PDK-1 Inhibitors

The serine/threonine protein kinase Akt forms a vital part of the PI3K pathway [8]. As Akt is frequently overactivated in solid organ cancers and implicated in tumour development and progression, Akt inhibitors have been developed for oncology trials [70]. Both allosteric and ATP-competitive inhibitors have been trialled. Significant toxicity has been observed and was sometimes treatment limiting, including diarrhoea, skin rash and hyperglycaemia.

3-phosphoinositide-dependent protein kinase 1 (PDK-1) is another essential regulator of protein kinases, including Akt [71]. PDK-1 inhibitors have demonstrated anticancer efficacy in cell lines but are not in routine clinical use [72]. PDK-1 and Akt inhibitors have not been reported for use in APDS. Toxicity from the inhibition of such vital components of a pathway required for a wide range of cellular processes is likely to preclude use in APDS. Furthermore, the downstream effects of PDK-1 inhibitors and Akt inhibitors on other kinases, such as SGK-1 [73], may result in metabolic complications such as hyperglycaemia, making these drugs inappropriate for long-term use in APDS.

## Monitoring Response to Treatment

The PI3K pathway is highly complex, involving feedback loops, cross-talk and compensatory pathways [56], many of which are likely to vary between individuals. Thus, measuring the effect of PI3K pathway inhibition at different points may be essential in optimising patient outcomes, and requires further study.

Historically our methods of measuring response to treatment in immunodeficiencies have been somewhat blunt. We record the frequency and severity of infections over time, measure sizes of lymph nodes and spleens, and monitor cytopenias. In the laboratory, we measure serum IgG concentrations after intravenous or subcutaneous immunoglobulin replacement. Given that the goal of treatment in APDS is subtle modulation of the PI3K pathway, preserving effective immune function while reducing lymphoproliferation and autoimmunity, and avoiding treatment toxicity in both the short and long term, more precision in assessing the degree of PI3K pathway inhibition and correlation with clinical response is required. Measurement of naïve T cell proportions and senescence patterns may be one approach, however detailed T cell phenotyping may be difficult to access in a routine diagnostic laboratory. It is encouraging that normalisation of endogenous serum immunoglobulin concentrations and lymphocyte counts has been observed after leniolisib [59, 60]. Sirolimus has also demonstrated clinical utility and improved immune cell subsets in some APDS patients, and further assessment and comparison of these responses would help guide treatment.

Measurement of the phosphorylation status of downstream effectors, such as Akt and S6 may be useful in determining the degree of PI3K pathway dysregulation, providing a qualitative rather than quantitative assessment [59]. Although this tool may help demonstrate treatment efficacy and correlation with clinical response in a research setting, it may be difficult to access in a therapeutic environment. As our understanding of APDS and the PI3K pathway expands, other biomarkers may emerge as relevant.

## Discussion

Much is made of the term “personalised medicine”, usually referring to the provision of tailored treatments to individual patients, and often involving the identification of a pathogenic genetic variant and subsequent targeted treatment. Clinical immunologists, however, practise personalised medicine for our patients every day. We taper corticosteroid doses according to an individual patient’s response (symptoms, signs, investigations), we determine choice of immunomodulatory medications based on comorbidities, lifestyle factors and patient preference. Sometimes, in consultation with the patient, we decide not to treat at all. The concept of personalised treatment in the management of autoimmune disease (classically viewed as immune overactivity) becomes intuitive for clinicians. However, historically immunodeficiency was less often viewed through the same lens, perhaps because until recently, treatment focused more on replacement of what was missing rather than downregulating what was overactive, unless overt autoimmunity was evident. We replenish gammaglobulins, we replace dysfunctional bone marrow with another, we prescribe prophylactic antibiotics to prevent infections. However, APDS is a disease of immune dysregulation, with as many features of immune overactivity as immunodeficiency, and perhaps our treatment approach now might have more in common with autoimmunity. In treating autoimmunity, we have a range of medications and dosages, and we more frequently tailor dosages to clinical response than to drug concentrations. In ANCA vasculitis, for example, we often use different medications to achieve “remission” than in the “maintenance” phase [74]. The identification of the molecular mechanisms underlying the pathogenesis of APDS has facilitated “personalised” treatment options, such as PI3Kδ inhibition rather than reliance on generic therapies, but even more personalisation of treatment, guided by assessment of degrees of PI3K pathway regulation, is likely to improve outcomes further.

Collaborative studies on APDS may help solve some of these unanswered questions. As mTORC1 integrates signals from such a wide range of inputs including cellular stress and DNA damage, do infections themselves drive further activation of the PI3K pathway in APDS? Furthermore, in APDS, T cells are skewed towards an effector phenotype, with an enhanced proliferative burst on exposure to antigen [3]. By extension, can the dose of a PI3Kδ inhibitor or other pathway modulator be reduced with time, when fewer infections result in reduced chronic signalling through receptors, and a reduced proportion of effector lymphocytes? Might treatment for “induction of remission” be different from “maintenance”, akin to management of many autoimmune diseases? Does improving enteropathy through other methods enhance the efficacy of sirolimus by altering its bioavailability and activation? What are the long-term risks of PI3K and mTOR inhibition, particularly in relation to metabolic effects and late onset immune related adverse effects? The clinical manifestations and immune cell abnormalities in APDS1 and APDS2 are similar but not identical [17]. Further study comparing treatment response in these patients would help determine whether different therapeutic strategies are required for these two groups.

In conclusion, management of APDS is likely to require nuanced modulation of the PI3K pathway as the consequences of overinhibition may be as deleterious as those associated with overactivity. Specific PI3Kδ inhibition has provided a promising new and targeted treatment option, however a greater understanding of the effects of PI3K pathway regulation at different points, more accurate measures of response, and longer term safety data are likely to improve our management of patients with this condition.

## Data Availability

Not applicable.

## References

[1] Maccari ME (2023). Activated phosphoinositide 3-kinase δ syndrome: update from the ESID registry and comparison with other autoimmune-lymphoproliferative inborn errors of immunity. J Allergy Clin Immunol.

[2] Angulo I (2013). Phosphoinositide 3-kinase δ gene mutation predisposes to respiratory infection and airway damage. Science.

[3] Lucas CL (2014). Dominant-activating germline mutations in the gene encoding the PI(3)K catalytic subunit p110δ result in T cell senescence and human immunodeficiency. Nat Immunol.

[4] Deau MC (2014). A human immunodeficiency caused by mutations in the PIK3R1 gene. J Clin Investig.

[5] Lucas CL (2014). Heterozygous splice mutation in PIK3R1 causes human immunodeficiency with lymphoproliferation due to dominant activation of PI3K. J Exp Med.

[6] Redenbaugh V, Coulter T (2021). Disorders related to PI3Kδ hyperactivation: characterizing the clinical and immunological features of activated PI3-kinase delta syndromes. Front Pediatr.

[7] Chen HH (2017). Immune dysregulation in patients with PTEN hamartoma tumor syndrome: analysis of FOXP3 regulatory T cells. J Allergy Clin Immunol.

[8] Okkenhaug K, Vanhaesebroeck B (2003). PI3K in lymphocyte development, differentiation and activation. Nat Rev Immunol.

[9] Chantry D (1997). p110delta, a novel phosphatidylinositol 3-kinase catalytic subunit that associates with p85 and is expressed predominantly in leukocytes. J Biol Chem.

[10] Okada T, Maeda A, Iwamatsu A, Gotoh K, Kurosaki T (2000). BCAP: the tyrosine kinase substrate that connects B cell receptor to phosphoinositide 3-kinase activation. Immunity.

[11] DulauFlorea AE (2017). Abnormal B-cell maturation in the bone marrow of patients with germline mutations in PIK3CD. J Allergy Clin Immunol.

[12] Okkenhaug K (2013). Signaling by the phosphoinositide 3-kinase family in immune cells. Annu Rev Immunol.

[13] Coulter TI (2017). Clinical spectrum and features of activated phosphoinositide 3-kinase δ syndrome: a large patient cohort study. J Allergy Clin Immunol.

[14] Elkaim E (2016). Clinical and immunologic phenotype associated with activated phosphoinositide 3-kinase δ syndrome 2: A cohort study. J Allergy Clin Immunol.

[15] Avery DT (2018). Germline-activating mutations in PIK3CD compromise B cell development and function. J Exp Med.

[16] Bloomfield M, Klocperk A, Zachova R, Milota T, Kanderova V, Sediva A (2021). Natural course of activated phosphoinositide 3-kinase delta syndrome in childhood and adolescence. Front Pediatr.

[17] Nguyen T et al (2023) Human PIK3R1 mutations disrupt lymphocyte differentiation to cause activated PI3Kδ syndrome 2. J Exp Med 220(6). 10.1084/jem.20221020 10.1084/jem.20221020 PMC10037341 36943234

[18] Bier J (2019). Activating mutations in PIK3CD disrupt the differentiation and function of human and murine CD4(+) T cells. J Allergy Clin Immunol.

[19] Edwards ESJ (2019). Activating PIK3CD mutations impair human cytotoxic lymphocyte differentiation and function and EBV immunity. J Allergy Clin Immunol.

[20] Wentink MWJ (2018). Exhaustion of the CD8(+) T cell compartment in patients with mutations in phosphoinositide 3-kinase delta. Front Immunol.

[21] Ruiz-García R (2018). Mutations in PI3K110δ cause impaired natural killer cell function partially rescued by rapamycin treatment. J Allergy Clin Immunol.

[22] Durandy A, Kracker S (2020). Increased activation of PI3 kinase-δ predisposes to B-cell lymphoma. Blood.

[23] Maccari ME (2018). Disease evolution and response to rapamycin in activated phosphoinositide 3-kinase δ syndrome: the european society for immunodeficiencies-activated phosphoinositide 3-kinase δ syndrome registry. Front Immunol.

[24] Tangye SG, Bier J, Lau A, Nguyen T, Uzel G, Deenick EK (2019). Immune dysregulation and disease pathogenesis due to activating mutations in PIK3CD-the goldilocks' effect. J Clin Immunol.

[25] Clayton E (2002). A crucial role for the p110delta subunit of phosphatidylinositol 3-kinase in B cell development and activation. J Exp Med.

[26] Okkenhaug K (2002). Impaired B and T cell antigen receptor signaling in p110delta PI 3-kinase mutant mice. Science.

[27] Lucas CL, Chandra A, Nejentsev S, Condliffe AM, Okkenhaug K (2016). PI3Kδ and primary immunodeficiencies. Nat Rev Immunol.

[28] Sogkas G, Fedchenko M, Dhingra A, Jablonka A, Schmidt RE, Atschekzei F (2018). Primary immunodeficiency disorder caused by phosphoinositide 3-kinase δ deficiency. J Allergy Clin Immunol.

[29] Cohen SB (2019). Human primary immunodeficiency caused by expression of a kinase-dead p110δ mutant. J Allergy Clin Immunol.

[30] Swan DJ (2019). Immunodeficiency, autoimmune thrombocytopenia and enterocolitis caused by autosomal recessive deficiency of PIK3CD-encoded phosphoinositide 3-kinase δ. Haematologica.

[31] Rodriguez R (2019). Concomitant PIK3CD and TNFRSF9 deficiencies cause chronic active Epstein-Barr virus infection of T cells. J Exp Med.

[32] Sharfe N (2018). Dual loss of p110δ PI3-kinase and SKAP (KNSTRN) expression leads to combined immunodeficiency and multisystem syndromic features. J Allergy Clin Immunol.

[33] Conley ME (2012). Agammaglobulinemia and absent B lineage cells in a patient lacking the p85α subunit of PI3K. J Exp Med.

[34] Dimitrova D (2022). International retrospective study of allogeneic hematopoietic cell transplantation for activated PI3K-delta syndrome. J Allergy Clin Immunol.

[35] Coulter TI, Cant AJ (2018). The treatment of activated PI3Kδ syndrome. Front Immunol.

[36] Rivalta B (2021). Case report: EBV chronic infection and lymphoproliferation in four APDS patients: the challenge of proper characterization, therapy, and follow-up. Front Pediatr.

[37] Laplante M, Sabatini DM (2012). mTOR signaling in growth control and disease. Cell.

[38] Nguyen LS (2019). Sirolimus and mTOR inhibitors: a review of side effects and specific management in solid organ transplantation. Drug Saf.

[39] Rae W (2016). Precision treatment with sirolimus in a case of activated phosphoinositide 3-kinase δ syndrome. Clin Immunol.

[40] Emerson JS, Lee EY, Berglund LJ (2021). Treatment of immune dysregulation due to a PTEN variant with sirolimus. J Clin Immunol.

[41] Shen G (2023). Precision sirolimus dosing in children: The potential for model-informed dosing and novel drug monitoring. Front Pharmacol.

[42] Utecht KN, Hiles JJ, Kolesar J (2006). Effects of genetic polymorphisms on the pharmacokinetics of calcineurin inhibitors. Am J Health Syst Pharm.

[43] Stenton SB, Partovi N, Ensom MH (2005). Sirolimus: the evidence for clinical pharmacokinetic monitoring. Clin Pharmacokinet.

[44] Paine MF (2002). Identification of a novel route of extraction of sirolimus in human small intestine: roles of metabolism and secretion. J Pharmacol Exp Ther.

[45] Baraldo M, Furlanut M (2006). Chronopharmacokinetics of ciclosporin and tacrolimus. Clin Pharmacokinet.

[46] Thirumaran RK (2012). Intestinal CYP3A4 and midazolam disposition in vivo associate with VDR polymorphisms and show seasonal variation. Biochem Pharmacol.

[47] Goyal RK (2013). Sirolimus pharmacokinetics in early postmyeloablative pediatric blood and marrow transplantation. Biol Blood Marrow Transplant.

[48] Schachter AD (2004). Short sirolimus half-life in pediatric renal transplant recipients on a calcineurin inhibitor-free protocol. Pediatr Transplant.

[49] Brattström C, Wilczek H, Tydén G, Böttiger Y, Säwe J, Groth CG (1998). Hyperlipidemia in renal transplant recipients treated with sirolimus (rapamycin). Transplantation.

[50] Groth CG (1999). Sirolimus (rapamycin)-based therapy in human renal transplantation: similar efficacy and different toxicity compared with cyclosporine. Sirolimus European Renal Transplant Study Group. Transplantation.

[51] Olbrich P (2016). Activated PI3Kδ syndrome type 2: two patients, a novel mutation, and review of the literature. Pediatr Allergy Immunol.

[52] Yap JY (2020). Everolimus-induced remission of classic Kaposi's sarcoma secondary to cryptic splicing mediated CTLA4 haploinsufficiency. J Clin Immunol.

[53] Klawitter J, Nashan B, Christians U (2015). Everolimus and sirolimus in transplantation-related but different. Expert Opin Drug Saf.

[54] Oleksak P, Nepovimova E, Chrienova Z, Musilek K, Patocka J, Kuca K (2022). Contemporary mTOR inhibitor scaffolds to diseases breakdown: A patent review (2015–2021). Eur J Med Chem.

[55] Voss MH et al. Phase 1 study of mTORC1/2 inhibitor sapanisertib (TAK-228) in advanced solid tumours, with an expansion phase in renal, endometrial or bladder cancer. Br J Cancer. 2020;123(11):1590–8. 10.1038/s41416-020-01041-x. 10.1038/s41416-020-01041-x PMC7686313 32913286

[56] Yang J, Nie J, Ma X, Wei Y, Peng Y, Wei X (2019). Targeting PI3K in cancer: mechanisms and advances in clinical trials. Mol Cancer.

[57] Alzahrani AS (2019). PI3K/Akt/mTOR inhibitors in cancer: at the bench and bedside. Semin Cancer Biol.

[58] Busaidy NL (2012). Management of metabolic effects associated with anticancer agents targeting the PI3K-Akt-mTOR pathway. J Clin Oncol.

[59] Rao VK (2017). Effective "activated PI3Kδ syndrome"-targeted therapy with the PI3Kδ inhibitor leniolisib. Blood.

[60] Rao VK (2023). A randomized, placebo-controlled phase 3 trial of the PI3Kδ inhibitor leniolisib for activated PI3Kδ syndrome. Blood.

[61] Cant AJ, Chandra A, Munro E, Rao VK, Lucas CL (2023). PI3Kδ pathway dysregulation and unique features of its inhibition by Leniolisib in activated PI3Kδ syndrome and beyond. J Allergy Clin Immunol: Pract.

[62] Begg M (2023). An open label trial of nemiralisib, an inhaled PI3 kinase delta inhibitor for the treatment of activated PI3 kinase delta syndrome. Pulm Pharmacol Ther.

[63] Diaz N (2020). Seletalisib for activated PI3Kδ syndromes: open-label phase 1b and extension studies. J Immunol.

[64] Lannutti BJ (2011). CAL-101, a p110delta selective phosphatidylinositol-3-kinase inhibitor for the treatment of B-cell malignancies, inhibits PI3K signaling and cellular viability. Blood.

[65] Gopal AK (2014). PI3Kδ inhibition by idelalisib in patients with relapsed indolent lymphoma. N Engl J Med.

[66] Furman RR (2014). Idelalisib and rituximab in relapsed chronic lymphocytic leukemia. N Engl J Med.

[67] Coutré SE (2015). Management of adverse events associated with idelalisib treatment: expert panel opinion. Leuk Lymphoma.

[68] Compagno M (2017). Phosphatidylinositol 3-kinase δ blockade increases genomic instability in B cells. Nature.

[69] Rao VK (2023). Interim analysis: open-label extension study of Leniolisib for patients with APDS. J Allergy Clin Immunol.

[70] Hua H, Zhang H, Chen J, Wang J, Liu J, Jiang Y (2021). Targeting Akt in cancer for precision therapy. J Hematol Oncol.

[71] Mora A, Komander D, van Aalten DM, Alessi DR (2004). PDK1, the master regulator of AGC kinase signal transduction. Semin Cell Dev Biol.

[72] Sestito S, Rapposelli S (2019). A patent update on PDK1 inhibitors (2015-present). Expert Opin Ther Pat.

[73] Lien EC, Dibble CC, Toker A (2017). PI3K signaling in cancer: beyond AKT. Curr Opin Cell Biol.

[74] Hellmich B (2023). EULAR recommendations for the management of ANCA-associated vasculitis: 2022 update. Ann Rheum Dis.

